# Role of Exosomal MicroRNAs and Their Crosstalk with Oxidative Stress in the Pathogenesis of Osteoporosis

**DOI:** 10.1155/2021/6301433

**Published:** 2021-07-19

**Authors:** Jun Lu, Yan Zhang, Jinqi Liang, Jiayu Diao, Peilong Liu, Hongmou Zhao

**Affiliations:** ^1^Foot and Ankle Surgery Department, Honghui Hospital of Xi'an Jiaotong University, Xi'an 710054, China; ^2^Cardiovascular Department, Shaanxi Provincial People's Hospital, Xi'an 710068, China

## Abstract

Osteoporosis (OP) is an aging-related disease involving permanent bone tissue atrophy. Most patients with OP show high levels of oxidative stress (OS), which destroys the microstructure of bone tissue and promotes disease progression. Exosomes (exos) help in the delivery of microRNAs (miRNAs) and allow intercellular communication. In OP, exosomal miRNAs modulate several physiological processes, including the OS response. In the present review, we aim to describe how exosomal miRNAs and OS contribute to OP. We first summarize the relationship of OS with OP and then detail the features of exos along with the functions of exo-related miRNAs. Further, we explore the interplay between exosomal miRNAs and OS in OP and summarize the functional role of exos in OP. Finally, we identify the advantages of exo-based miRNA delivery in treatment strategies for OP. Our review seeks to improve the current understanding of the mechanism underlying OP pathogenesis and lay the foundation for the development of novel theranostic approaches for OP.

## 1. Introduction

Osteoporosis (OP) is a disease caused by abnormal bone metabolism [1] due to the dysfunction and abnormal differentiation of osteoclasts and osteoblasts [2, 3]. Patients with OP show increased osteoclast differentiation and decreased osteoblast differentiation [3]. The loss of bone density in OP weakens bone structure, leading to fractures. OP can also result in pain and other complications, reducing a patient's ability to engage in activities of daily living. The mechanism underlying OP remains unclear, although recent studies have been successful in exploring its biology in further detail [4].

Oxidative stress (OS), a risk factor for OP, has received increasing attention in recent years. Studies have shown that OS can inhibit the differentiation of osteoblasts in bone marrow and stimulate the differentiation of osteoclasts, promoting the occurrence and development of OP [5, 6]. OS occurs as a result of the constant production of reactive oxygen species (ROS), such as superoxide anions (O^2-^), hydrogen peroxide (H_2_O_2_), hydroxyl radicals (OH^−^), and other free radicals, during metabolic processes in the human body [7]. Superoxide dismutase, glutathione peroxidase, catalase, and reduced glutathione constitute the main antioxidant defense systems in humans [8]. In addition, dietary antioxidants can complement the endogenous antioxidant system [9].

Under normal circumstances, there is a balance between ROS production and the body's antioxidant defense [10]. Controlled production of free radicals by normal osteoclasts can accelerate the destruction of calcified tissue and contribute to bone reconstruction [11, 12], which is very important for the growth and development of bones and for fracture repair. However, factors such as smoking, aging, and estrogen deficiency can disrupt the redox balance, resulting in OS [13]. OS causes extensive oxidative damage to cells—it inhibits the differentiation, growth, and proliferation of most cells and accelerates cell aging and death via the activation of several signal transduction pathways, such as the NF-*κ*B, MAPKs, p53, and HSF pathways [14, 15].

Therefore, OS also affects bone remodeling.

Bone remodeling is coordinated and regulated by osteoclasts and osteoblasts, and studies suggest that these two cell types communicate with each other [16, 17]. In addition to substantial evidence demonstrating that osteoblasts guide osteoclast bone resorption, studies have also shown that osteoclasts regulate osteoblast bone formation through direct cell-to-cell contact and cytokine-mediated indirect contact [18]. However, it is unclear whether any other “paracrine” pathways mediate the communication between osteoclasts and osteoblasts [19].

Recently, exosomal miRNAs have been shown to regulate several physiological processes, including the OS response, in OP [20–23]. Our review seeks to enhance the understanding of the mechanism underlying OP pathogenesis and to lay the foundation for novel theranostic approaches for OP.

## 2. OS and OP

OS is caused by the build-up of free radicals, including those generated as a result of inflammation and mitochondrial dysfunction [24]. ROS are the primary contributors to the aggravation of OS and tissue damage [25]. ROS production and clearance are a dynamic process that is affected by multiple factors. Under normal conditions, appropriate levels of ROS are required to maintain certain signaling pathways, enhance cell proliferation, and regulate cell metabolism [26]. However, when the normal redox state of cells is disturbed, peroxides and free radicals are produced. This causes damage to all cell components, including proteins, lipids, and DNA, leading to cellular toxicity [7, 8, 27].

OP is a systemic skeletal disorder [28, 29] that results from reduced maximum bone mass levels and elevated bone loss [30]. Given the involvement of abnormal metabolism in OP, this disease is particularly sensitive to OS, and the relationship between the two has therefore gathered significant attention. Moreover, OS is known to contribute to diseases of bone metabolism, especially OP, as elevated OS is often observed in the bone tissue of OP patients. Therefore, OS may be a potential target for the treatment of OP [31, 32].

Previous findings demonstrate the detrimental effect of OS on bone health [33–35]. ROS are thought to affect the bone environment via two modes of action. Primarily, ROS may potentiate the responsiveness of osteoclast precursors to RANKL, and secondarily, it may induce the production of additional osteoclastogenic cytokines such as IL-1, IL-6, and IL-7 [36, 37]. Furthermore, OS may also affect the function of osteoblasts. Recent studies have shown that ROS decrease the life span of osteoblasts in osteoporotic mice [38]. Interestingly, both endogenous and dietary antioxidants were found to mitigate and delay bone loss in a number of animal studies. Moreover, various forms of vitamin E have been found to prevent the reduction in trabecular number and bone volume in osteoporotic mice [39, 40]. In addition, a number of epidemiological studies have demonstrated that bone mass density has a positive and inverse relationship with OS biomarkers and antioxidant status, respectively.

## 3. Exosomes (Exos) and Exo-Associated miRNAs

### 3.1. Biological Characteristics of Exos

Exos are extracellular vesicle-like substances with a diameter of 30–150 nm and are found in almost all functional and nonfunctional biological fluids. Exos are a part of a large family of membrane vesicles, which also includes extracellular microvesicles (100–350 nm) [41] and apoptotic vesicles (500–1000 nm) [42, 43]. Exos are thought to be involved in many biological processes and play an important role in cell-to-cell communication [44–46]. Most cells release exos into the extracellular environment after plasma membrane fusion [47–49]. The discovery of exos dates back to 1983, when researchers cultured reticulocytes to track the movement of transferrin receptors from the plasma membrane to reticulocytes. Surprisingly, they found that the tagged transferrin receptors were taken up by reticulocytes and then reassembled into small vesicles within reticulocytes. At first, it was thought that these vesicles would be destroyed by lysosomes inside the cell and then expelled out of mature red blood cells, but the actual functions of these vesicles were subsequently discovered.

**Figure 1:**
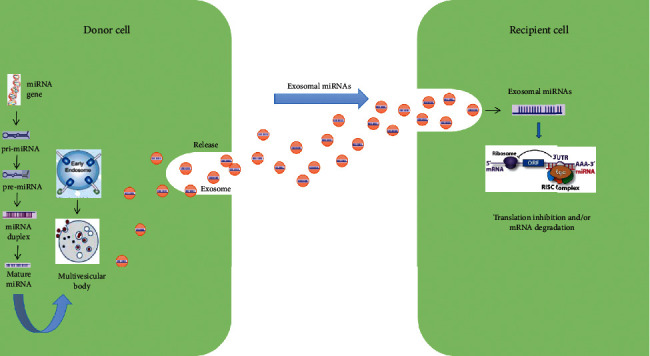
Exosomal miRNA biogenesis and interaction with target cells. A variety of miRNAs are contained in exos and delivered to target cells.

Lipids and proteins are the main active constituents of exos, and a variety of nucleic acids, including mRNA, miRNAs, and other noncoding RNAs, have also recently been found to be present [50]. When exos are secreted and released into body fluids, they can reach target cells. After being taken up by target cells, exos can release active RNAs and therefore play a role in the subsequent regulatory processes (Figure 1).

The mechanism underlying the identification and internalization of exos is a key focus of investigation. According to evidence from recent studies, exo uptake is specific. Moreover, exos are adept at delivering their contents to specific acceptor cells. For instance, exos released by fibroblasts (NIH-3T3 cells) are capable of delivering antagomir-188 to mesenchymal stem cells (MSCs) in a bone-targeted manner [51]. Moreover, MSC-derived exos can promote angiogenesis and osteogenesis by delivering exosomal miR-29a [52]. However, the main pathway governing the delivery of exos to specific target cells remains unclear, although there are some hypotheses to explain this phenomenon. One hypothesis suggests that target cells identify and engulf exos based on their size and membrane components [53, 54]. For example, CD47 on the exosomal membrane may prevent the endocytosis of exos by monocytes and macrophage [55]. Additionally, CD11a and CD54—which are present on the surface of dendritic cells—and CD9 and CD81—which are present on the surface of exos—may promote exosomal targeting to dendritic cells as well as their engulfment [56]. Another hypothesis suggests that the molecular cargo carried by exos itself targets exos to specific cells, but evidence supporting this postulation is lacking. Given the extensive potential of exos in targeted therapy, the molecular and cellular mechanism via which they maintain their specificity warrants additional investigation.

### 3.2. Exosomal miRNAs

#### 3.2.1. Characteristics

miRNAs are small noncoding RNAs (17–24 in length) that bind to the 3′UTR or open reading frame of target mRNAs and regulate posttranscriptional gene silencing [57]. The miRNAs present in exos can be delivered to neighboring or distant cells, where they exert regulatory effects. Exosomal miRNAs play a key role in bone metabolism-related disease progression [20, 58, 59], and three potential mechanisms have been implicated in their pathogenic role. First, miRNAs are thought to target mRNAs of regulatory genes and suppress their translation or promote protein degradation. Second, miRNAs are thought to contribute to OP pathogenesis by directly binding to toll-like receptors or regulating their transcriptional expression. Finally, miRNAs are considered to cause a miRNA formation disorder [60]. Moreover, the current evidence indicates a strong relationship between miRNAs and OS.

#### 3.2.2. Role of Exosomal miRNAs in OP

*(1) Novel Avenues for Gene Therapy*. Exos can deliver functional miRNAs and have thus been used to develop exo-based targeted gene therapy [61]. Owing to their high biosafety and immune evasion abilities, exos have great potential as miRNA vectors [62]. Currently, exo-based miRNA delivery systems are being explored using animal models [63]. Duan et al. confirmed the efficacy of miR-140 delivery using engineered exos for osteoarthritis therapy [64]. The expression of miRNAs changes across the different phases of OP, and some miRNAs play a role in OP progression [65]. Consequently, the control of miRNA expression using exos could be a feasible approach for gene therapy in cases of OP.

*(2) Exo-Based Cell–Cell Communication*. Chemical receptor-mediated communication is the most well-documented form of cell–cell communication [66]. Exos and their transport across different cells have widened our understanding of cell–cell communication. miRNAs, one of the most important elements present in exos, have been demonstrated to participate in cell–cell communication [67]. For an example, the circulating exosomal miR-20b-5p that is released from cells in patients with diabetic foot ulcers is known to transmit functional information via paracrine secretion and regulate diabetic wound healing—a process dependent on various cells, including vascular endothelial cells and fibroblasts [68]. Furthermore, M2 macrophages can deliver miR-5106-containing exos to bone marrow MSCs (BMSCs) and regulate protein expression in these cells [69]. Given the importance of BMSCs in bone remodeling, their role in the onset and progression of OP is unsurprising. Taken together, this evidence may provide novel avenues for examining the association of exosomal miRNAs with OP.

## 4. Crosstalk between Exosomal miRNAs and OS in OP

Both OS and exo-derived miRNAs play crucial roles in the occurrence of OP. Interestingly, OS regulates many miRNAs, and conversely, miRNAs also regulate genes participating in the OS response [70]. A recent study found that OS upregulates the expression of miR-34a in exosomes derived from muscles, and this miRNA then induces cellular senescence in bone stem cells. In C2C12 myoblasts, the overexpression of exosomal miR-34a suppresses Sirt1 mRNA and protein expression [71]. miR-34a induces senescence in vascular smooth muscle cells and cardiomyocytes and promotes cardiac fibrosis [72, 73]. It has been suggested that with aging and increased exposure to inflammatory factors and ROS, both of which increase OS, miR-34a is upregulated as a consequence of p53 activation, which occurs in cases of sepsis, injury, and inflammation [74].

miR-182-5p was previously reported to inhibit osteoblast proliferation and differentiation by targeting Foxo1 [75], and miR-183-5p was found to be elevated during cellular senescence after exposure to OS [76]. Davis et al. found that bone-derived exos are capable of impairing MSC proliferation and inducing bone stem cell senescence. Moreover, miR-183-5p, an exosomal miRNA, was demonstrated to be a significant active contributor to this regulation. Furthermore, *in vitro* assays based on H_2_O_2_-induced OS have indicated that H_2_O_2_ treatment increases the abundance of miR-183-5p in bone-derived exos in MSCs and that H_2_O_2_ levels in the bone marrow microenvironment increase with age [76].

## 5. Role of Exosomal miRNAs in OP

Owing to the biological characteristic of exos, exosomal miRNAs can exist stably in the body and can remain stable for 48 hours at 4°C *in vitro* [77]. These special features allow exos to play a regulatory role in certain diseases. The functional effects of exosomal miRNAs in OP have been well documented (Table 1) [52, 78–81]. Li et al. reported that exos derived from MSCs can effectively ameliorate the development of OP, and exosomal miR-186 participates in this regulatory process [81]. Moreover, a recent study demonstrated that exosomal miR-1263 derived from human umbilical cord MSCs can inhibit osteoblast apoptosis and that nanomaterials loaded with miR-1263 may be ideal alternatives for the treatment of bone resorption disorders [80]. Song et al. assessed upregulated miR-155 levels in exos derived from vascular endothelial cells and suggested that the exos and the exosomal miR-155 may serve as bone-targeting and nontoxic nanomedicines for the treatment of OP [79]. Furthermore, Xu et al. reported that miR-31a-5p levels are significantly elevated in exos from aging BMSCs. These levels contribute to age-related changes in the bone marrow microenvironment and affect osteoblastic and osteoclastic differentiation [78]. In addition, exosomal miR-29a was recently demonstrated to inhibit OP progression by promoting osteogenesis and angiogenesis [52]. Therefore, exosomal miRNAs play important roles in the development of OP.

## 6. Exo-Based miRNA Delivery for OP Treatment

The blood–brain barrier (BBB) is one of the most challenging hindrances against treatment strategies for bone remodeling diseases, delaying the development of novel clinical agents [82]. Exos can traverse the BBB, and studies have reported that exos have many additional advantages as delivery vehicles for drugs and nanoparticles [83], including a high delivery efficiency, good biocompatibility, and efficient management of the inflammatory response [84]. The exo-mediated delivery of miRNAs for OP treatment has become the focus of recent research. MSC-derived exos have been found to be effective in delivering functional miRNAs that promote osteogenic differentiation and inhibit the development of OP [78–80]. Recent studies have demonstrated that local or systemic application of exos has potential as a treatment option for OP [85–88]. Current research on exos has advanced beyond the observation stage, and convincing experimental results have been obtained. However, from the perspective of clinical applications, these results should be interpreted with caution. First, there is currently no widely applicable method for exo isolation and validation. Existing isolation techniques lead to the inevitable mixing of nonexosomal components, such as lipoproteins, proteins, viruses, and bacteria, with exos isolated from different specimens [89–91]. In addition, the standards for separation are not uniform, and the different equipment used across different laboratories may lead to further differences and inconsistencies, which will eventually lead to different findings [92, 93]. The techniques used for the characterization of exos are also different, and their accuracy varies too [94]. Finally, a variety of methods are used to determine the concentration of exos, including simple quantitative protein determination and nanoparticle tracking analysis, and a wide variety of units are used for quantitation [95, 96].

Owing to these problems, it is necessary to standardize sample collection methods and methods for separation, characterization, and quantitation in order to facilitate the collection of reliable and replicable data across different laboratories and research areas. Furthermore, researchers need to be aware of the challenges involved in the experimental procedures put forth in the recent guidelines from the International Society of Extracellular Vesicles. Given that a gold standard for exo isolation and characterization has not been established, researchers should perform thorough literature reviews to identify the most suitable isolation method for their research.

## 7. Conclusion and Perspectives

In summary, exosomal miRNA-mediated OS affects osteoblasts, osteoclasts, and the bone matrix, promoting the development of OP. Antioxidants have the potential to inhibit OS. When the antioxidant balance in the body is disrupted, exogenous antioxidants can help in preventing or postponing the development of OP. However, current research in this field is limited. Therefore, it is necessary to conduct in-depth basic and clinical studies to clarify the role of OS and exosomal miRNAs in the occurrence and development of OP and to develop novel and improved treatments for this disease.

## Figures and Tables

**Table 1:** The role of exosomal miRNAs in osteoporosis.

Exosome source	RNA extraction	RNA identification	Exosomal miRNA(s)	Regulatory role	Reference
Human bone marrow mesenchymal stem cells	Exosome extraction kits (QIAGEN, Germany)	RT-qPCR	miR-186	Ameliorate	Li et al., 2021, [81]
Human umbilical cord mesenchymal stem cell	Trizol reagent	RT-qPCR	miR-1263	Ameliorate	Yang et al., 2020, [80]
Vascular endothelial cell	Trizol reagent	RT-PCR	miR-155	Ameliorate	Song et al. 2019, [79]
Bone marrow stromal cells of aging mice	Trizol reagent	RT-qPCR	miR-31a-5p	Aggravate	Xu et al., 2017, [78]
Bone marrow mesenchymal stem cells	Trizol reagent	RT-qPCR	miR-29a	Ameliorate	Lu et al., 2020

## Data Availability

The data used to support the findings of this study are included within the article.
